# How Reliable is eHealth information on dental care in head and neck cancer? A quality evaluation

**DOI:** 10.1186/s12903-026-08488-z

**Published:** 2026-04-29

**Authors:** Felix Marschner, Ludwig Runkel, Maximilian Range, Clemens Lechte, Annette Wiegand

**Affiliations:** Department of Preventive Dentistry, Periodontology and Cariology, University Medical Center Göttingen, Robert-Koch-Str. 40, Göttingen, 37075 Germany

**Keywords:** Head and neck cancer, Dental care, eHealth, Digital media, Website, Information quality

## Abstract

**Background:**

Given the increasing reliance on online health information, this study aimed to systematically assess the quality of German-language eHealth information on head and neck cancer (HNC) related dental care.

**Methods:**

German-language websites were searched via Google.de, Bing.de/Yahoo.de, and DuckDuckGo.com in February 2025. German-language Youtube-videos were searched in March 2025. Websites were assessed across 4 domains: technical/functional aspects (LIDA-instrument), readability (Flesh-reading-ease-score), comprehensiveness (structured checklist), and quality and risk of bias (DISCERN-instrument). Differences between domains were tested using the Friedman test. Group differences among provider types were examined with one-way ANOVA or Kruskal-Wallis tests. YouTube-videos were assessed for comprehensiveness, viewers’ interaction, and viewing rate. The Wilcoxon rank-sum test compared comprehensiveness between Youtube-videos and websites.

**Results:**

A total of 134 eligible websites and 26 YouTube-videos were included. 63.4% of the websites were operated by private dental practices. All four domains differed significantly from each other (*p* < 0.001). Websites from private and corporate dental practices or private hospital groups showed significantly lower scores in technical/functional aspects compared with websites from dental societies, regulatory bodies, public institutions, or insurance companies. Overall readability was poor, with the highest scores observed for institutional websites (median 49.0) and the lowest for private practices (median 38.0). Comprehensiveness of patient-oriented information was low, especially among corporate dental practices and private hospital groups (median 5.0). Quality of consumer health information was highest for commercial or non-profit information services (median 29) and lowest for private and corporate dental practices (median 23.0). Only 19.2% of YouTube-videos originated from private dental practices, and exhibited low viewer interaction (median 0.9). No significant difference in comprehensiveness was observed between websites and YouTube-videos (*p* = 0.924).

**Conclusions:**

German-language eHealth information on dental care in HNC is generally of low quality. This study highlights the need for standardized, reliable, and patient-oriented online resources to support oral health and quality of life in HNC patients.

**Supplementary Information:**

The online version contains supplementary material available at 10.1186/s12903-026-08488-z.

## Background

Dental care plays a crucial role in head and neck cancer (HNC) management and can significantly contribute to a reduction of complications, for instance by screening for and treatment of dental foci prior to (chemo)radiotherapy, the fabrication of retraction and fluoride trays for using during and after radiotherapy, and regular follow-up dental care [1, 2]. The treatment of oral side effects resulting from cancer therapy can lead to long-term improvement of both oral health and the oral health-related quality of life of HNC patients [3, 4].

Given the burden of HNC, patients may deprioritize oral health. Therefore, providing high-quality health information is crucial to raise awareness of the importance of oral health and potential complications associated with therapy. The internet is becoming an important resource for patients to access eHealth information, especially on websites and YouTube videos [5, 6]. For HNC patients, the internet is the second most preferred source of information after face-to-face consultations with healthcare professionals [7]. Written content is generally favored over videos due to its perceived usefulness, and prior eHealth use is often motivated by a desire to learn about potential treatment side effects [8]. It is essential that this information is accessible for patients in a way that is understandable, reliable, and complete [9]. Considering that patients rely on this information, trust and accuracy are essential requirements for eHealth content [5, 6]. Previous studies have shown that the quality and reliability of patient-related eHealth information often fail to meet these criteria [9–12]. It has been shown that YouTube contains a large volume of healthcare-related content, some of which is inaccurate or deceptive [6]. Whether this is also the case for content related to HNC in the dental context remains unclear at present. Nevertheless, misleading health information may lead to delayed dental pretreatment, inadequate oral hygiene, and refusal of necessary dental interventions [11].

Furthermore, the visibility and dissemination of online content are often influenced by factors such as search engine optimization, the popularity of videos or websites, and commercial interests, rather than the quality or scientific accuracy of the information [13, 14]. In recent years, search engines like Google have applied stricter scrutiny to health and medical websites, for content with potentially health-related impact, higher standards are imposed regarding authority, quality, and trustworthiness [15]. However, studies show that widely shared or popular eHealth content often exhibits quality deficiencies, such as missing sources, lack of balance, or insufficient transparency regarding commercial interests [16]. For patients, including those with HNC, this means they may be more likely to encounter easily accessible or visually appealing content rather than evidence-based, complete, and independent information. This increases the risk of misinformation, delayed or foregone necessary dental interventions, and suboptimal management of side effects from (chemo-)radiotherapy. Accordingly, eHealth information must be not only accessible but also developed, maintained, and disseminated according to clear standards of quality, transparency, and evidence, particularly when it serves vulnerable groups such as HNC patients.

Systematic evaluations of German web-based eHealth information for HNC patients in the dental context, comprising both websites and YouTube videos, are currently lacking. This study, therefore, aims to assess that quality and to explore practice-related factors influencing the content provided by private dental practices. It was hypothesized that practice-related factors (e.g., location, practice size, and dentists’ demographics) would be associated with differences in website quality among private dental practices.

## Methods

This study adhered to the Enhancing Transparency in Reporting the Synthesis of Qualitative Research [17] statement. As all data used in this research are publicly accessible and non-sensitive, the research ethics committee of the University Medical Center Göttingen (15/2/25) has confirmed that no ethical approval is required.

### Eligibility criteria

Freely accessible German-language websites and YouTube videos providing patient-oriented information regarding HNC and related dental issues were included. Eligible sources comprised dental care providers and health-related institutions involved in patient care or patient education, including private and corporate dental practices, private practices, private hospital groups, public dental clinics, dental schools, dental societies, regulatory bodies, public institutions, insurance companies, and commercial or non-profit information services.

Excluded were websites and YouTube videos that did not primarily provide patient-oriented dental or healthcare information. This included content providers, particularly those associated with dental laboratories, dental supply and material companies, manufacturers of oral hygiene products, as well as advertisements, articles, books, research institutions not focused on patient care, and forums or blogs operated by non-dentists. Additionally, YouTube videos longer than 15 min or without audio and subtitles were excluded.

### Search strategy

Between February 15 and 18, 2025, a systematic search of websites was conducted in 3 electronic search engines: google.de, bing.de/yahoo.de, and duckduckgo.com. These electronic search engines were selected based on their market share in Germany and collectively accounted for over 95% of German internet search traffic at the time of the search [18]. Eight search terms were employed across the electronic search engines, utilizing different German synonyms for dentists (in both male and female forms), dentists, dental practice, alongside various terms related to HNC in professional and layperson language (Supplementary Table A.1). The same search strategy was applied on YouTube to identify videos on March 13, 2025.

### Website selection

All queries were executed on a computer running Windows 10 Home (Microsoft Inc.), and Firefox version 135.0 (Mozilla Foundation) with internet access from Germany. All searches were performed using the default settings of the electronic search engines. Before each search, the browser’s cache, cookies, and history were completely cleared. Advertisements and/or sponsored websites displayed at the top of search results were disregarded. Only websites presented by the electronic search engines as the “most relevant” were considered.

Websites were initially assessed based on the search result snippets to determine their relevance to the inclusion criteria. Those meeting the criteria were selected for full screening. If the content was found to contain irrelevant information, it was excluded from consideration. This was followed by the removal of duplicate entries. The website search and inclusion process were managed by one author (L.R.) and verified by another author (F.M.). Any disagreements between reviewers were resolved through discussion.

### Data extraction

Data extraction from the included websites and YouTube video was performed independently by two authors (L.R. and M.R.) using a pilot-tested spreadsheet (Supplementary Table A2, and Supplementary Table A3). Any disagreements were resolved through repeated data extraction and consultation with another author (F.M.). Extracted information included the following categories: name of content provider, URL, country of the content provider, location of the provider (i.e., rural [< 5,000 inhabitants], town [< 100,000 inhabitants], or city [≥ 100,000 inhabitants]), and type of provider (i.e., private and corporate dental practices, private hospital groups, university hospitals, dental societies, regulatory bodies, public institutions, insurance companies, and commercial or non-profit information services).

For websites and YouTube videos published by private dental practices, additional data were extracted: practice setting (i.e., single practitioner or multiple dentists); sex (i.e., male, female, or mixed [in case of multiple dentists]); dental society memberships; and years of examination (averaged in case of multiple dentists). Additionally, video-specific data were extracted from YouTube videos: upload date; duration (in minutes); number of likes; and number of comments.

### Outcomes

Quality of patient-oriented eHealth information for patients with HNC on websites was systematically assessed across the following four domains:

1. technical and functional aspects, assessed by the LIDA validation instrument (version 1.2; Minervation) to evaluate the accessibility (1.1), usability (1.2), and reliability (1.3) [19] (score range: 0 to 96, with higher scores indicating better technical and functional aspects).
2. readability, assessed by the Flesch reading-ease score (FRES) [20], adapted for the German language [21] (score range: 0 to 100, with higher scores indicating easier-to-read text).
3. comprehensiveness of information, evaluated by a structured checklist, regarding clinical signs (3.1), preventive measures and/or early detection (3.2), preventive measures to avoid oral complications (3.3), and dental treatment options (3.4) for HNC patients (score range: 0 to 30, with higher scores indicating better comprehensiveness of information). The checklist was developed by two expert clinicians (F.M. and A.W.).
4. quality and risk of bias, assessed by the DISCERN instrument [22] to evaluate the reliability (4.1) and quality (4.2) of written health information for consumers (score range: 16 to 80, with higher scores indicating better reliability).

Domains 1, 3, and 4 were evaluated independently by two authors (L.R. and M.R.). Any disagreements were resolved through discussion. While domains 1, 3 and 4 were manually scored, domain 2 was evaluated using an automated, freely available online tool calculating the FRES [23]. Additionally, for YouTube videos, viewing rates were calculated using the number of views relative to the days since upload, while viewers’ interaction rates were determined using engagement metrics such as likes and comments in relation to total views, as described in previous studies [24, 25]. These calculations were independently reviewed by two authors (F.M. and L.R.).

### Statistical analysis

Descriptive statistics of domains included the median, interquartile range (IQR), and range between maximum and minimum scores. Differences between quality domains were assessed using Friedman test followed by Conover test post-hoc test. Differences across provider types were examined using one-way ANOVA (for normally distributed data) with Bonferroni post-hoc test, and Kruskal-Wallis test with Dunn-Bonferroni post-hoc test (for non-normally distributed data). Websites provided by private dental practices, the impact of practice-specific variables on quality domains and overall quality scores were assessed using generalized linear modeling (GLM). The comprehensiveness of information (domain 3) was compared between websites and YouTube videos using the Mann-Whitney-U test. Interrater agreement among researchers was assessed using intraclass correlation coefficient (ICC 2,1) analysis [26]. The level of significance was set to *p*-value < 0.05. Statistical analysis was performed using JASP (Version 0.19.3 Apple Silicon).

## Results

### Website and YouTube search

**Fig. 1:**
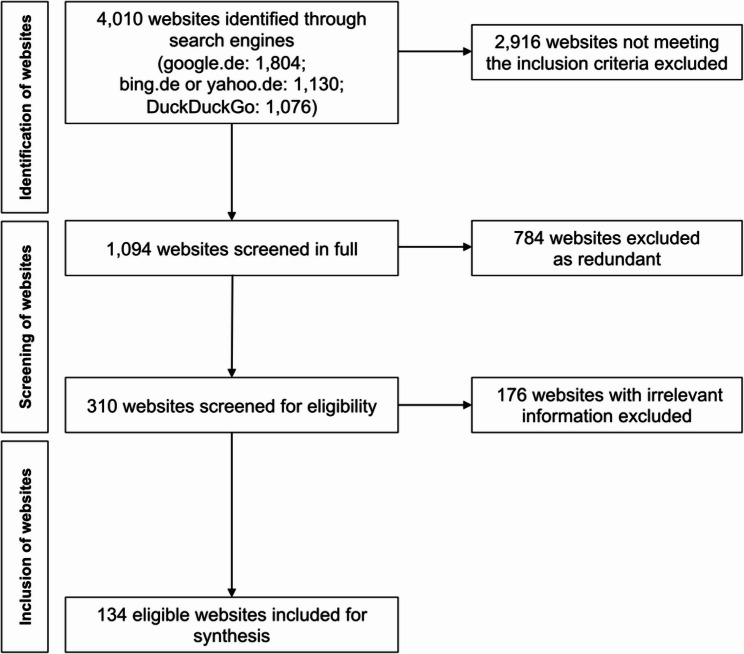
Flowchart of systematic website search and evaluation

A total of 4,010 websites were identified through search engines. 134 eligible websites and 26 YouTube videos were included for synthesis. A flowchart of the systematic website search is shown in Fig. 1. The characteristic features of the included websites and YouTube videos are presented in Supplementary Table A.2 and Supplementary Table A.3. The interrater reliabilities across all domains indicated an excellent agreement [27], technical and functional aspects: 0.985; comprehensiveness of information: 0.977 (websites), 0.980 (YouTube videos); quality and risk of bias: 0.996.

### Websites

Approximately two thirds of the included websites were operated by private dental practices, or private practices, while the remaining websites (36.6%) were run by other providers. 92.5% of the websites were based in Germany. Detailed characteristics of included websites are shown in Table 1.

**Table 1:** Characteristics of included German-language websites and YouTube videos

Characteristics	Websites (*n* = 134), *n* (%)	YouTube videos (*n* = 26), *n* (%)
*Country*		
Germany	124 (92.5)	22 (84.6)
Austria	4 (3.0)	N/A
Switzerland	4 (3.0)	3 (11.5)
Hungary	1 (0.75)	N/A
Belgium	1 (0.75)	N/A
Canada	N/A	1 (3.9)
*Content provider*		
Private dental practice, or private practice^a^	85 (63.4)	5 (19.2)
Corporate dental practice, or private hospital group	23 (17.2)	5 (19.2)
University hospital	7 (5.2)	5 (19.2)
Dental society, regulatory body, public institution, or insurance company	15 (11.2)	3 (11.6)
Information service	4 (3.0)	8 (30.8)

*N/A* not applicable

^a^ two private radiation therapy practices reported on dental care services on their websites

Among the websites provided by private dental practices (*n* = 83) most of them were located in cities (*n* = 53). Regarding private dental practice demographics, 63.9% had employed multiple dentists and 34.9% worked in a mixed-gender setting. All other recorded private dental practice demographics are presented in Table 2.

**Table 2:** Dentists’ demographics of included German-language websites and YouTube videos operated by private dental practices

Characteristics	Websites (*n* = 83), *n* (%)	YouTube videos (*n* = 5), *n* (%)
*Country*		
Germany	77 (92.8)	4 (80.0)
Austria	3 (3.6)	N/A
Switzerland	2 (2.4)	1 (20.0)
Hungary	1 (1.2)	N/A
*Location*		
Rural (< 5,000 inhabitants)	7 (8.4)	N/A
Town (< 100,000 inhabitants)	23 (27.7)	1 (20.0)
City (≥ 100,000 inhabitants)	53 (63.9)	4 (80.0)
*Practice setting*		
Single dentist	30 (36.1)	1 (20.0)
Multiple dentists	53 (63.9)	4 (80.0)
*Sex*		
Male	33 (39.8)	1 (20.0)
Female	21 (25.3)	1 (20.0)
Mixed	29 (34.9)	3 (60.0)
Dental society membership		
Yes	54 (65.0)	3 (60.0)
No, or unknown	29 (35.0)	2 (40.0)
Dentists’ year of examination, mean (SD)^a^	2002.7 (9.2)	2004.5 (6.1)

*SD* standard deviation, *N/A* not applicable

^a^ in the case of multiple dentists, the years of examination were averaged, if available

**Fig. 2:**
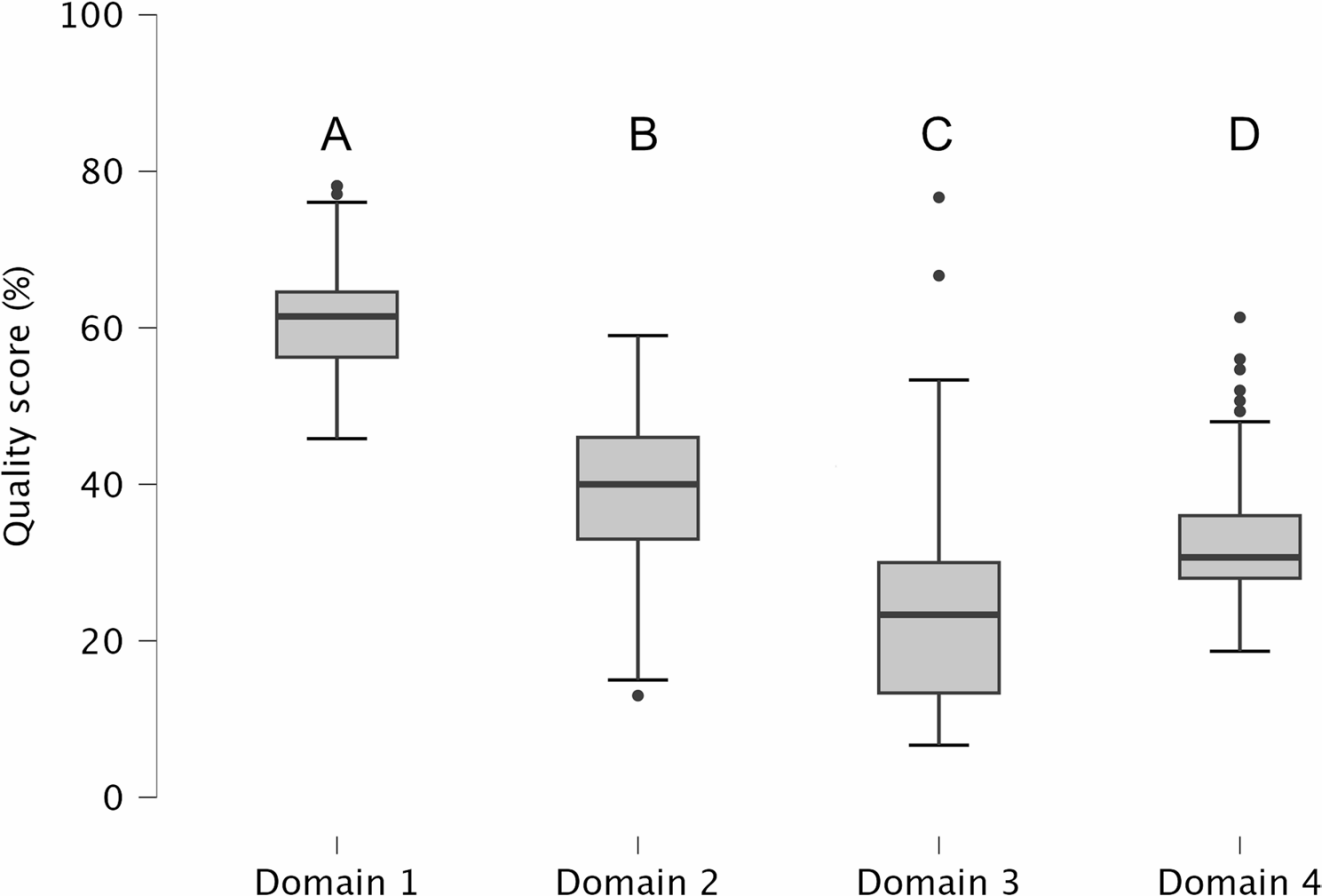
For all websites, quality scores of each domain (relative percentage of maximum possible score sum). Domain 1: technical and functional aspects were evaluated using the LIDA validation instrument (version 1.2; Minervation) [19]; domain 2: readability was evaluated using the Flesch reading-ease score [20], adapted for the German language [21]; domain 3: comprehensiveness of information; domain 4: quality and risk of bias was assessed with the DISCERN instrument [22]; outliers are marked with a dot (·); different capital letters indicate significant differences between domains (*p*-value < 0.05), Friedman test followed by the Conover test post-hoc test

All four domains differed significantly across all included websites (*p* < 0.001, Fig. 2). The quality across all domains varied among different content providers (Table 3). For technical and functional aspects, all analyzed websites demonstrated a consistently moderate level of quality (reference LIDA score 50 to 90) [19, 28]. Websites from private practices and corporate dental practices, or private hospital groups achieved significantly lower LIDA scores (median 57.0, IQR 8.0 and median 57.0, IQR 8.5, respectively) than websites from dental societies, regulatory bodies, public institutions, or insurance companies (median 68.0, IQR: 10.0). For FRES, all websites were difficult to read [29]. Dental societies, regulatory bodies, public institutions, or insurance companies showed the highest scores (median 49.0), while private practices had the lowest readability (median 38.0). Especially, the comprehensiveness of patient-oriented eHealth information regarding HNC and related dental issues was low. The lowest comprehensiveness of information score was observed in corporate dental practices, or private hospital groups, with a median score of 5.0 (IQR: 3.5). Quality and reliability of written health information for consumers, assessed using the DISCERN instrument, was highest for information services (median 29.0, IQR: 8.3), and lowest for private dental practice, or private practice and corporate dental practice, or private hospital group (median 23.0, IQR: 7.0 and median 23.0, IQR 6.0, respectively). Overall, all website providers showed poor (reference DISCERN score 27 to 38) or very poor (reference DISCERN score 16 to 26) quality and reliability [30].

**Table 3:** Quality scores of all domains across website providers

Outcomes	Website provider					*p*-value
	Private dental practice, or private practice	Corporate dental practice, or private hospital group	University hospital	Dental society, regulatory body, public institution, or insurance company	Information service	
LIDA validation instrument [19]^a^ Median (IQR) Range	57.0 (8.0)^B^ 44.0–74.0	57.0 (8.5)^BC^ 51.0–67.0	61.0 (2.5)^ABC^ 58.0–67.0	68.0 (10.0)^A^ 57.0–75.0	63.5 (5.5)^AB^ 62.0–75.0	< 0.001*
Flesch reading-ease score [20, 21]^b^ Median (IQR) Range	38.0 (11.0)^B^ 13.0–59.0	41.0 (15.5)^AB^ 21.0–59.0	37.0 (6.0)^AB^ 28.0–42.0	49.0 (6.5)^A^ 30.0–58.0	38.0 (10.3)^AB^ 21.0–44.0	0.013*
Comprehensiveness of information^c^ Median (IQR) Range	6.0 (4.0)^BC^ 2.0–14.0	5.0 (3.5)^C^ 2.0–12.0	9.0 (2.5)^AB^ 4.0–16.0	10.0 (4.5)^A^ 3.0–23.0	11.5 (1.8)^A^ 10.0–14.0	< 0.001*
DISCERN instrument [22]^d^ Median (IQR) Range	23.0 (7.0)^A^ 14.0–39.0	23.0 (6.0)^AC^ 14.0–31.0	24.0 (3.5)^A^ 21.0–30.0	28.0 (7.5)^AB^ 19.0–46.0	29.0 (8.3)^A^ 22.0–37.0	0.018*

^a^ score range: 0 to 96, with higher scores indicating better technical and functional aspects

^b^ score range: 0 to 100, with higher scores indicating easier-to-read text

^c^ score range: 0 to 30, with higher scores indicating better comprehensiveness of information

^d^ score range: 16 to 80, with higher scores indicating better reliability; IQR, interquartile range

* statistical significant (*p*-value < 0.05), ANOVA with Bonferroni post-hoc test or Kruskal-Wallis tests with Dunn-Bonferroni post-hoc test; within each row different capital letters indicate significant differences between website providers

GLM analyses assessing associations between quality domains and dentists’ demographic characteristics on websites of private dental practices revealed no significant improvement over the respective null models (likelihood ratio test, all *p* > 0.05), indicating limited explanatory power of the included predictors. Domain-specific analyses showed weak associations for the year of dental examination and technical and functional aspects (B = 0.3, 95% confidence interval [CI]: 0.0 to 0.5, *p* = 0.041), practices with multiple dentists in readability scores (B = − 9.8, 95% CI: −17.8 to − 1.8, *p* = 0.020), and urban practice locations in quality scores (B = 5.0, 95% CI:1.0 to 9.0, *p* = 0.018), indicating that later examination years were associated with slightly higher technical and functional aspects scores, practices with multiple dentists had lower readability scores, and urban practices had moderately higher quality scores.

### YouTube videos

The majority of assessed YouTube videos were provided in Germany (84.6%), and only 19.2% originated from private dental practices. Further information of characteristics of the included YouTube videos is presented in Table 1. Detailed information of YouTube videos provided by private dental practice is shown in Table 2. Viewers’ interaction rate was low (median 0.9, IQR: 1.0) (Table 4). The comprehensiveness of patient-oriented information regarding HNC and related dental issues did not differ statistically significantly between all included websites, with a median score of 6.5 (IQR: 6.0), and YouTube videos, with a median score of 7.0 (IQR: 5.0) (*p* = 0.924).

**Table 4:** Characteristics of all included German-language YouTube videos (*n* = 26)

Characteristics	Median (IQR)	Range
Age of video (years)	3.0 (2.8)	1.0-13.1
Duration (minutes)	5.2 (4.1)	1.1–13.4
Number of views (*n*)	1,277 (9,106.3)	7-404,993
Number of likes (*n*)	6.5 (25.5)	0–2,013
Number of comments (*n*)	0.5 (3.0)	0-287
Viewers’ interaction^a^ (%)	0.9 (1.0)	0.0-6.2
Viewing rate^b^ (%)	135.7 (386.2)	1.0–75,137.8

*IQR* interquartile range

^a^ viewers’ interaction was calculated using the formula described in previous studies [24, 25]

^b^ viewing rate was calculated using the formula described in previous studies [24, 25]

## Discussion

This study analyzed the quality of German web-based eHealth information on HNC and related dental issues across websites and YouTube videos. In terms of technical and functional characteristics, most websites reached moderate levels of performance [19, 28]. More structured organizational content providers, such as dental societies or public institutions, achieved higher scores, while private and corporate practices scored lower. These differences may reflect variations in IT support, editorial resources, or website maintenance capabilities [31]. The included websites were generally difficult to read, far below the recommended FRES ≥ 70 (6th-8th grade reading level) for patient materials [29]. Websites from dental societies, regulatory bodies, public institutions, or insurance companies, scored slightly higher, likely due to professional editing or standardized patient communication guidelines. Particularly, the comprehensiveness of information regarding clinical signs, preventive measures and/or early detection, preventive measures to avoid oral complications, and dental treatment options for HNC patients was limited with the lowest score for eHealth information from websites provided by corporate dental practice, or private hospital groups. This may be partially explained by differences in organizational priorities and available resources for patient education [32]. Furthermore, resource allocation strategies in larger practice networks or management organizations may differ, which warrants further investigation [32]. Regarding the quality of written health information for consumers, the quality and reliability was poor to very poor [30]. Our findings are in accordance with previous studies on eHealth information [33–35], which underscore that information quality is a widespread challenge. Analyses of dentists’ demographic characteristics revealed no significant improvement in model fit, suggesting limited explanatory value of these factors. However, minor domain-specific trends were observed: more recent dental examinations were associated with slightly higher technical and functional aspects scores, practices with multiple dentists tended to have lower readability scores, and urban practices showed somewhat higher quality scores. These findings suggest that, while small patterns exist, demographic characteristics alone are insufficient to explain variability in website quality. Other determinants, such as practice policies or content management strategies, may play a more substantial role and warrant further investigation. Only a few YouTube videos were available (*n* = 26) with low comprehensiveness of information. Interestingly, no significant differences were observed between websites and YouTube videos, which contrasts with previous findings suggesting that video-based resources often exhibit lower low comprehensiveness of information than written content [36, 37]. A possible explanation is the limited number of relevant videos, most originating from institutional content providers, reducing heterogeneity in quality. The low viewer interaction rate (median 0.9%) further suggests limited patient engagement and visibility, possibly because the topic is not consistently present in public discourse. Additionally, the low comprehensiveness of the identified videos may have further contributed to reduced viewer engagement, as incomplete or insufficiently detailed content is likely to discourage sustained interaction and sharing behavior.

Our results align with recent studies indicating that eHealth information frequently suffers from deficiencies in readability, comprehensiveness, and overall quality [24], especially in the oncological context [10, 28, 33, 34, 38]. However, the applied quality framework reflects a clinical perspective and may not fully capture consumer-perceived quality, as patients have been shown to prioritize visual design and navigability over content completeness, even though comprehensive content may be particularly relevant for supporting informed health-related decision-making [39]. As the internet is the second most preferred source of information after direct consultation with healthcare professionals [7], it is crucial to ensure that eHealth information is accurate and understandable. Misinformation or incomplete guidance can lead to misconceptions about dental care, hinder rehabilitation, and increase healthcare costs. In this context, standardized quality seals, such as the Health On the Net Foundation code, which certifies reliable health information websites, structured readability checks, and input from interdisciplinary expert panels could greatly improve credibility and usability [40]. However, it should be acknowledged that patients represent a heterogeneous group with varying literacy levels, informational needs, and preferences. Standardization efforts should therefore be accompanied by flexible, tailored formats to ensure information is accessible and meaningful to diverse patient populations [41, 42]. Collaboration with patient organizations and advocacy groups should also be prioritized, as trusted messengers within patient communities may be more effective in disseminating accurate, accessible, and culturally appropriate information than institutional providers alone. National dental societies and patient organizations should also expand their efforts by creating centralized, multimedia resources tailored specifically to the oral care needs of HNC patients.

The main limitation of our study was the cross-sectional nature of the systematic search. The search was conducted in February and March 2025, making the results time-sensitive. Website updates or removals may alter the findings over time. Another limitation of this study was its focus on German-language websites and YouTube videos, which therefore may not generalize to other linguistic or cultural contexts or health care systems. Furthermore, the search was conducted without a double search procedure, which may have introduced a degree of variability in the retrieved results and should be considered in future studies. Additionally, the search strategy was limited to search engines, which may not capture all pathways through which patients access eHealth information (e.g., social contacts, or healthcare professionals). The comprehensiveness checklist used in this study was developed based on expert clinical experience and reflects routine care of HNC patients in daily practice. However, patients were not formally involved in its development, which may limit its alignment with patient-perceived information needs. Readability assessment was limited to the FRES [20, 21, 23], which captures syntactic complexity but not other relevant dimensions of health literacy, such as understandability or actionability [43]. Future studies should consider multidimensional tools such as the Patient Education Materials Assessment Tool [43]. Finally, YouTube metrics such as “number of likes” or “number of comments” provide limited insight into actual patient comprehension or behavioral impact [37, 44]. Besides these limitations, our study also has several strengths. Websites were identified through a systematic web search using three different electronic search engines (google.de, bing.de/yahoo.de, and duckduckgo.com) following a reproducible search strategy that included eHealth information from various content providers. The use of different German synonyms for dentists and dental practices, alongside various terms related to HNC in both professional and layperson language, was another strength. Furthermore, the use of validated assessment tools (LIDA [19], FRES [20, 21, 23], and DISCERN [22]) and the excellent interrater reliability across all domains strengthen the robustness of our findings.

Future studies should examine how patients with HNC perceive, evaluate, and use dental-related eHealth information, combining content analyses with qualitative insights from users. In particular, future eHealth information should be developed using co-design approaches that involve patients as active partners, as evidence suggests that patient involvement in the design phase substantially improves the relevance and effectiveness of eHealth information [45]. Close collaboration between oncologists, dental professionals, patient advocacy groups, and digital media experts could support the development of accessible and evidence-based eHealth information.

## Conclusions

German-language eHealth information on HNC and related dental issues across websites and YouTube videos is generally of low quality, lacking comprehensiveness and reliability.

Given the critical importance of oral health for the prognosis and quality of life of HNC patients, systematic efforts are needed to develop standardized, high quality, and patient-oriented eHealth information.

## Supplementary Information

Supplementary Material 1.

## Data Availability

All data analyzed during this study are available from the corresponding author upon reasonable request.

## Abbreviations

FRES: Flesch reading-ease score
GLM: Generalized linear modeling
HNC: Head and neck cancer
ICC: Intraclass correlation coefficient
IQR: Interquartile range
N/A: Not applicable
SD: Standard deviation
