# Supplementary material for: How Reliable is eHealth information on dental care in head and neck cancer? A quality evaluation
Source: BMC Oral Health. 2026 Apr 29;26:814. doi: 10.1186/s12903-026-08488-z (PMC13157662; doi:10.1186/s12903-026-08488-z)
Supplement: Supplementary file 1 — Supplementary Material 1. [file 12903_2026_8488_MOESM1_ESM.docx]

**Supplementary material**

Supplementary Table A.1. German search terms used and number of results (n) for each electronic search engine.

| **Search term^a^** | **Google: google.de**  **(n)** | **Bing or Yahoo: bing.de/yahoo.de (n)** | **DuckDuckGO: duckduckgo.com (n)** |
| --- | --- | --- | --- |
| (Zahnärztin OR Zahnarzt OR Zahnärzte OR Zahnarztpraxis) Kopf-Hals-Tumorerkrankungen | 199 | 30 | 30 |
| (Zahnärztin OR Zahnarzt OR Zahnärzte OR Zahnarztpraxis) Kopf-Hals-Tumore | 216 | 86 | 73 |
| (Zahnärztin OR Zahnarzt OR Zahnärzte OR Zahnarztpraxis) Kopf-Hals-Karzinome | 193 | 29 | 16 |
| (Zahnärztin OR Zahnarzt OR Zahnärzte OR Zahnarztpraxis) Kopf-Hals-Krebs | 232 | 36 | 29 |
| (Zahnärztin OR Zahnarzt OR Zahnärzte OR Zahnarztpraxis) Rachenkrebs | 244 | 205 | 187 |
| (Zahnärztin OR Zahnarzt OR Zahnärzte OR Zahnarztpraxis) Mundhöhlenkrebs | 226 | 255 | 238 |
| (Zahnärztin OR Zahnarzt OR Zahnärzte OR Zahnarztpraxis) Mundkrebs | 254 | 263 | 294 |
| (Zahnärztin OR Zahnarzt OR Zahnärzte OR Zahnarztpraxis) Zungenkrebs | 240 | 226 | 209 |

^a^ Search terms represent different German synonyms for male and female dentists, dentists, dental practice, head and neck cancers, head and neck tumors, head and neck carcinomas, head and neck cancer, throat cancer, oral cavity cancer, oral cancer, tongue cancer

Supplementary Table A.2. Characteristics of included websites with eHealth information for HNC patients in dental context.

| **No.** | **Name of content provider** | **URL** | **Country** | **Location^a^** | **Type of provider** | **Practice setting (sex)** | **Society membership^b^** | **Year of examination^b^** |
| --- | --- | --- | --- | --- | --- | --- | --- | --- |
| 1 | Die Zahnärzte – Dr. Edzard Fink & Peter Knor GbR | <https://www.za-fink.de/leistungen/prophylaxe-erhaltungstherapie/mundgesundheit-kopf-hals-tumoren> | Germany | Bremen (city) | Private dental practice | Multiple dentists (three male dentists) | N/R | 1984, 1993, N/R |
| 2 | Die Zahnärzte Radevormwald Dr. Irga & Irga | <https://www.zahnaerzte-rade.de/aktuelles/> | Germany | Radevormwald (town) | Private dental practice | Multiple dentists (one female, and one male dentist) | N/R | N/R |
| 3 | Dr. Butz und Partner Zahnärzte | <https://www.zahnarzt-fuenfhoefe.de/leistungen/oralchirurgie/schleimhautveraenderungen-muenchen/> | Germany | Munich (city) | Private dental practice | Multiple dentists (three female, and four male dentists) | DGI, DGZMK, GZFA, DGET, BDO, DGMKG | 2007, 2004, 2007, 2006, 2022, 2013, 2011 |
| 4 | Dr. Dimitrios Papavasileiou & Dr. Philipp Raab GbR, Privatpraxis für Zahnheilkunde | <https://zahnarzt-lehel.de/behandlung/mundkrebsvorsorge/> | Germany | Munich (city) | Private dental practice | Multiple dentists (two male dentists) | DGPro | N/R, N/R |
| 5 | Dr. Dr. Thea Lingohr MSc., Zahnärztin und Oralchirurgin | <https://dr-lingohr.de/zahnarztpraxis-koeln-suedstadt-bayenthal/> | Germany | Cologne (city) | Private dental practice | Multiple dentists (four female dentists) | N/R | 2007, 2012, 2012, 2017 |
| 6 | Dr. Heide Kettler-Nölke, Praxis für Zahngesundheit | <https://www.zahnarztpraxis-hamburg-niendorf.de/1-285-509-Wei%C3%9Fe-Flecken-im-Mund-k%C3%B6nnen-zu-Mundh%C3%B6hlenkrebs-f%C3%BChren.html> | Germany | Hamburg (city) | Private dental practice | Single dentist (one female dentist) | DGZMK, DGI, NLI, DGCZ, AG Keramik, FVDZ | N/R |

Supplementary Table A.2. Continued.

| No. | Name of content provider | URL | Country | Location^a^ | Type of provider | Practice setting (sex) | Society membership^b^ | Year of examination^b^ |
| --- | --- | --- | --- | --- | --- | --- | --- | --- |
| 7 | Dr. Marcus Flach - Zahnarzt Wuppertal | <https://dr-flach.de/behandlungen/sonstige-behandlungen/mundschleimhauterkrankungen/> | Germany | Wuppertal (city) | Private dental practice | Single dentist (one male dentist) | PZVD, DGZMK | 1991 |
| 8 | Dr. med. dent. Alexandra Thaler Zahnarztpraxis | <https://www.dr-alexandra-thaler.de/mundgesundheit-bei-krebsbehandlung/> | Germany | Nuremberg (city) | Private dental practice | Multiple dentists (two female dentists) | DGZMK | N/R, N/R |
| 9 | Dr. med. dent. Christian Bergmeier | <https://www.zahnarztpraxis-bergmeier.de/behandlungsspektrum/mundkrebs.html> | Germany | Ingolstadt (city) | Private dental practice | Multiple dentists (two female, and one male dentist) | N/R | N/R, N/R, N/R |
| 10 | Dr. med. dent. David Bosman | <https://www.zahnarzt-bosman.de/zahnmedizin/mundhoehlenkrebs.html> | Germany | Aachen (city) | Private dental practice | Single dentist (one male dentist) | DGÄZ, DGET | N/R |
| 11 | Dr. med. dent. Nora Joos | <https://www.zahnarztpraxis-joos.de/mund-undrachenraumkrebsvorsorge> | Germany | Berlin (city) | Private dental practice | Multiple dentists (one female, and one male dentist) | DGParo, DGZMK | N/R, N/R |
| 12 | Dr. med. dent. Axel Posorski | <https://www.dr-posorski.de/krebsfrueherkennung-hamburg/> | Germany | Hamburg (city) | Private dental practice | Single dentist (one male dentist) | DGI, DGZMK | 1992 |
| 13 | Dr. med. Dr. med. dent. Volker Zieglowski | <https://www.fine-face.de/startseite.html> | Germany | Mayen (town) | Private dental practice | Single dentist (one male dentist) | DGMKG, DGI, DGZS, DGZMK, DGZH, MEG, DGBT, DGKiZ | N/R |

Supplementary Table A.2. Continued.

| No. | Name of content provider | URL | Country | Location^a^ | Type of provider | Practice setting (sex) | Society membership^b^ | Year of examination^b^ |
| --- | --- | --- | --- | --- | --- | --- | --- | --- |
| 14 | Dr. Mirjam Grüttner-Schroff, Zahnärztin & Zahntechnikerin | <https://gruettner-schroff-zahnarzt.de/leistungen/mund-und-kiefergesundheit/> | Germany | Bonn (city) | Private dental practice | Multiple dentists (two female dentists) | N/R | 2004, 1995 |
| 15 | Dr. Schlendermann, Zahnarztpraxis | <https://www.dr-schlendermann.de/content/aktuell.php> | Germany | Lüneburg (town) | Private dental practice | Multiple dentists (two male dentists) | DGParo, BDIZ EDI, DGZMK, DGI | 2018, N/R |
| 16 | elements – ZAHNMEDIZIN, Anastasia Giakmoglidou | <https://elements-zahnmedizin.de/krebsvorsorge/> | Germany | Esslingen (town) | Private dental practice | Multiple dentists (two female dentists) | DEGUZ | 2004, 2012 |
| 17 | Fachzahnarzt für Oralchirurgie, Dr. med. dent. Matthias Polta | <https://oralchirurgie-koblenz.de/patienten-info/krebsvorsorge/> | Germany | Koblenz (city) | Private dental practice | Multiple dentists (two male dentists) | BDIZ EDI, BDO, DGZI, DGZMK, FVDZ | 2006, N/R |
| 18 | Fachzahnarztpraxis hillebrand; Dr. med. dent. Udo Hillebrand | <https://www.zahnarzt-haltern.de/mundhoehlenkrebs-vorsorge/> | Germany | Haltern am See (town) | Private dental practice | Multiple dentists (one female, and two male dentists) | DGZMK | N/R, N/R, N/R |
| 19 | feinwerk Zahnmedizin, Dr. Tatjana Hoffmann | <https://feinwerk-zahnmedizin.de/zahnmedizin/mundkrebsvorsorge/> | Germany | Bietigheim-Bissingen (town) | Private dental practice | Multiple dentists (two female dentists) | DGParo, DGZMK, DGCZ, DGI | 2012, 2021 |

Supplementary Table A.2. Continued.

| No. | Name of content provider | URL | Country | Location^a^ | Type of provider | Practice setting (sex) | Society membership^b^ | Year of examination^b^ |
| --- | --- | --- | --- | --- | --- | --- | --- | --- |
| 20 | Florian Kubitzek – Facharzt für Mund-Kiefer-Gesichtschirurgie | <https://www.mkg-kubitzek.de/sport-gesundheit/betreuung-von-krebspatienten> | Germany | Munich (city) | Private dental practice | Single dentist (one male dentist) | N/R | N/R |
| 21 | Gelencsér Dental Kft., Ungarn | <https://zahnarzt-ungarn-heviz.de/dental-magazin/zahnbehandlung-nach-tumoroesen-erkrankungen> | Hungary | Hévíz (rural) | Private dental practice | Multiple dentists (six female, and twelve male dentists) | N/R | 2003, 2012, 2020, 1992, 2004, 2005, 2011, 2001, 2012, 1999, 2018, 2014, 2018, N/R, N/R, N/R, N/R |
| 22 | Gemeinschaftspraxis (GbR), Die Zahnarztpraxis am Rathaus, Dr. med. dent. Ines Goch, Dr. med. dent. Constanze van Betteray, MSc. | <https://www.zahnarztpraxisamrathaus.de/dental-lexikon/mundhoehlenkrebs-karzinom/> | Germany | Langenfeld (town) | Private dental practice | Multiple dentists (two female dentists) | DGParo, DGZS, DGZMK, FVDZ, DGET | 2003, 2004 |
| 23 | Gülseren Köksal, Fachärztin für Mund- Kiefer- und Gesichtschirurgie | <https://kieferchirurgie-pankow.de/bei-schleimhautveraenderungen-zahnarzt-aufsuchen/> | Germany | Berlin (city) | Private dental practice | Single dentist (one female dentist) | N/R | N/R |

Supplementary Table A.2. Continued.

| No. | Name of content provider | URL | Country | Location^a^ | Type of provider | Practice setting (sex) | Society membership^b^ | Year of examination^b^ |
| --- | --- | --- | --- | --- | --- | --- | --- | --- |
| 24 | mainzahn – Zentrum für Zahngesundheit, Dr. Malte Otte MSc. | <https://mainzahn.com/uncategorized/kann-ein-zahnarzt-sie-auf-mundkrebs-untersuchen/> | Germany | Würzburg (city) | Private dental practice | Multiple dentists (three female, and three male dentists) | N/R | 2005, N/R, N/R, N/R, N/R, N/R |
| 25 | MASCHA & ROSSKOPF; Dr. Dr. Frank Mascha & Dr. Fabian Roßkopf | <https://www.mascha-rosskopf.de/leistungen/tumorchirurgie> | Germany | Burgberg im Allgäu (rural) | Private dental practice | Multiple dentists (two male dentists) | BDO, DGI, ITI, Zahnärztlicher Arbeitskreis Kempten | 2010, 2014 |
| 26 | MKG Niederrhein Dr. Dr. Brüggenolte Dr. Dr. Bergmann | <https://mkg-niederrhein.de/> | Germany | Geldern (town) | Private dental practice | Multiple dentists (two male dentists) | N/R | 2010, 2009 |
| 27 | Nicolas Meyer-Stolten Zahnmedizin & Prophylaxe | <https://zahnarztpraxis-meyer-stolten.de/patienteninfo/1-7-508-Gef%C3%A4hrliche-Wechselbeziehungen-von-Allgemeingesundheit-und-Mundgesundheit.html/> | Germany | Hamburg (city) | Private dental practice | Single dentist (one male dentist) | DGÄZ, VDZE, DGZMK | 1992 |
| 28 | Praxis Dr. med. Stephan Anders | <https://www.dr-anders.de/leistungen/krebsvorsorge.php> | Germany | Bad Aibling (town) | Private dental practice | Multiple dentists (two male dentists) | N/R | N/R, N/R |
| 29 | Praxis Dr. Stefan Mauß | <https://www.praxis-dr-mauss.de/unser-behandlungsspektrum/mundkrebsfr%C3%BCherkennung/> | Germany | Hannover (city) | Private dental practice | Single dentist (one male dentist) | ZVFFM, Royal Society of Medicine | 1991 |

Supplementary Table A.2. Continued.

| No. | Name of content provider | URL | Country | Location^a^ | Type of provider | Practice setting (sex) | Society membership^b^ | Year of examination^b^ |
| --- | --- | --- | --- | --- | --- | --- | --- | --- |
| 30 | Praxis für sanfte Zahnheilkunde; Dr. Andrea Sandner | <https://www.zahnaerztin-dr-sandner.de/behandlungsbegleitung.html> | Germany | Koblenz (city) | Private dental practice | Single dentist (one female dentist) | DGZMK, DGL, DGZH, MEG, DGM, GSAAM | N/R |
| 31 | Praxis für Zahngesundheit und Prophylaxe; Dr. Frank Priepke und Dr. Ann Lau | <https://www.zahnarztpraxis-berlin-treptow.de/1-285-509-Wei%C3%9Fe-Flecken-im-Mund-k%C3%B6nnen-zu-Mundh%C3%B6hlenkrebs-f%C3%BChren.html> | Germany | Berlin (city) | Private dental practice | Multiple dentists (one female, and two male dentists) | DEGUZ | 2004, 1996, 1994 |
| 32 | Praxis für Zahngesundheit, Dr. Reinhard Uhlig | <https://www.zahngesundheit-hamburg.de/1-285--Mundgesundheit.html> | Germany | Hamburg (city) | Private dental practice | Single dentist (one male dentist) | N/R | 1973 |
| 33 | Praxis für Zahnheilkunde & Implantologie, Dr. Hartmut Bongartz MSc MSc & Kollegen | <https://www.zahnarzt-drbongartz.de/technik/mundkrebs-frueherkennung> | Germany | Mönchen-gladbach (city) | Private dental practice | Multiple dentists (two female, and two male dentists) | BDO, DGParo, DGZMK, BDIZ EDI | N/R, 2015, 2015, 2014 |
| 34 | Praxis für Zahnheilkunde und Prophylaxe, Carsten Pook | <https://www.zahnarzt-holm.de/1-285-509-Wei%C3%9Fe-Flecken-im-Mund-k%C3%B6nnen-zu-Mundh%C3%B6hlenkrebs-f%C3%BChren.html> | Germany | Holm (rural) | Private dental practice | Single dentist (one male dentist) | DGZMK | 1994 |

Supplementary Table A.2. Continued.

| No. | Name of content provider | URL | Country | Location^a^ | Type of provider | Practice setting (sex) | Society membership^b^ | Year of examination^b^ |
| --- | --- | --- | --- | --- | --- | --- | --- | --- |
| 35 | Praxis für Zahnheilkunde, Implantologie und Prophylaxe; Dr. med. dent. Rolf Mäder | <https://www.zahnarzt-maeder.de/mundkrebsvorsorge.html> | Germany | Dettingen an der Erms (town) | Private dental practice | Multiple dentists (one female, and one male dentist) | DGZI, DZOI | 1985, 2015 |
| 36 | Praxis für Zahnmedizin Dr. Langowsky | <https://dr-langowsky.de/oralchirurgie/krebsvorsorge/> | Germany | Radeberg (town) | Private dental practice | Single dentist (one female dentist) | DGZMK | 1992 |
| 37 | Praxis Olivier | <https://www.zahnmedizin-herne.de/news/mundkrebs-erkennen-und-verhindern/> | Germany | Herne (city) | Private dental practice | Single dentist (one male dentist) | DGAZ, DGZMK, DGI, ÖGP, DGParo | 2008 |
| 38 | Praxis-Klinik Wuppertal, Dr. med. univ. Dr. med. dent. Friedrich Scheerer | <https://mkg-wuppertal.de/krebs-im-mund/> | Germany | Wuppertal (city) | Private dental practice | Multiple dentists (two male dentists) | DGI, DGMKG, DEGUM, GTÜM, DGMM, DGHNOKC | N/R, N/R |
| 39 | Privatpraxis für Zahnheilkunde; Dr. med. dent. Thomas Schröder | <https://www.privatpraxis-stuttgart.de/zahnarzt-leistungen/mundkrebs-vorsorge-frueherkennung/> | Germany | Stuttgart (city) | Private dental practice | Single dentist (one male dentist) | DGI, DGZMK, AKI, DGAAM, GAK | 1993 |

Supplementary Table A.2. Continued.

| No. | Name of content provider | URL | Country | Location^a^ | Type of provider | Practice setting (sex) | Society membership^b^ | Year of examination^b^ |
| --- | --- | --- | --- | --- | --- | --- | --- | --- |
| 40 | Privatpraxis für Zahnmedizin Dr. Kent und Kollegen | <https://www.dr-kent.de/leistungen/krebstherapiebegleitende-zahnmedizin/> | Germany | Bochum (city) | Private dental practice | Multiple dentists (three male dentists) | DGZMK, DGParo, DGÄZ, DGFDT, APW | 1994, 2008, 2001 |
| 41 | Dental Concept, Praxis für Zahn-, Mund- und Kieferheilkunde, Dr. med. dent. C. Schwermann, Dr. med. dent. A. Bareksei | <https://www.zahnarzt-voerde.de/mundkrebs.php> | Germany | Voerden (town) | Private dental practice | Multiple dentists (two female, and one male dentists) | DAA, DGCZ, DGZMK, DGZS, DZV | N/R, N/R, N/R |
| 42 | DENTAL CORNER – Zahnarzt Frankfurt Dr. med. dent. Susanne Magin | <https://www.dental-corner.de/kopf-hals-tumor-wie-der-zahnarzt-helfen-kann/> | Germany | Frankfurt (city) | Private dental practice | Single dentist (one female dentist) | N/R | 1997 |
| 43 | die Strahlen Therapeuten GbR | <https://www.diestrahlentherapeuten.de/behandlungsfelder/tumorerkrankungen/kopf-hals-tumore-1/> | Germany | Coburg (town) | Private practice | N/A | N/A | N/A |

Supplementary Table A.2. Continued.

| No. | Name of content provider | URL | Country | Location^a^ | Type of provider | Practice setting (sex) | Society membership^b^ | Year of examination^b^ |
| --- | --- | --- | --- | --- | --- | --- | --- | --- |
| 44 | Radioonkologie im Vosspalais, Privatpraxis, Prof. Dr. med. S. Marnitz, Prof. Volker Budach, Frau Dr. med. Maria Steingräber | <https://www.radiotherapy.care/portfolio-items/kopf-hals-tumoren/> | Germany | Berlin (city) | Private practice | N/A | N/A | N/A |
| 45 | Ron Tehsmer, Zahnarztpraxis | <https://cityzahn.de/leistungen-angebote-zahngesundheit/krebsvorsorge-frueherkennung-mundhoehlenkrebs/> | Germany | Hamburg (city) | Private dental practice | Multiple dentists (one female, and one male dentist) | DGET, VDZE, DGZMK, DGZ | 1996, 2017 |
| 46 | TEAM 15 - Praxis für Zahnmedizin & Kompetenzzentrum für Implantologie; Dr. Frederic Hermann | <https://www.team15.ch/krebsvorsorge/> | Switzer-land | Zug (town) | Private dental practice | Multiple dentists (one female, and five male dentists) | SSO | 2002, 1988, 1994, 2022, 1999, 1999 |
| 47 | Thorsten Mack, Zahnarzt | <https://www.zahnarzt-trochtelfingen.de/leistungsspektrum/krebsvorsorge/> | Germany | Trochtel-fingen (town) | Private dental practice | Single dentist (one male dentist) | N/R | 2004 |
| 48 | WEISS32; Dr. stom./Univ. Novi Sad Sebastijan Mormer - Zahnarzt | <https://www.weiss32.de/leistungen/vorsorge/krebsvorsorge/> | Germany | Stuttgart (city) | Private dental practice | Multiple dentists (four female, and three male dentists) | N/R | N/R, N/R, N/R, N/R, N/R, N/R, N/R |

Supplementary Table A.2. Continued.

| No. | Name of content provider | URL | Country | Location^a^ | Type of provider | Practice setting (sex) | Society membership^b^ | Year of examination^b^ |
| --- | --- | --- | --- | --- | --- | --- | --- | --- |
| 49 | zahn&Art (GbR), Dr. Sam Blum, Cátia Santos | <https://www.zahnarzt-berlin-mitte-zahnundart.de/leistungen/krebsvorsorge> | Germany | Berlin (city) | Private dental practice | Multiple dentists (two female, and one male dentist) | DGZS, APW, DGI, DGZMK | 2002, N/R, 2017 |
| 50 | Zahnarzt Dr. S. Buff und Dr. S. Nemitz | <https://zahnarzt-praxis-wiesbaden.de/tumorchirurgie-operationen-in-der-mundhoehle/> | Germany | Wiesbaden (city) | Private dental practice | Multiple dentists (three female, and three male dentists) | BDO, DGZMK | 1993, 1997, N/R, N/R, N/R, N/R |
| 51 | Zahnarzt und Fachzahnarzt für Oralchirurgie (Zahnärztliche Chirurgie); Dr. Thomas Wiener | <https://www.kieferchirurgie-salzburg.at/wissenswert/ist-mundkrebs-vorsorge-sinnvoll/> | Austria | Salzburg (city) | Private dental practice | Multiple dentists (one female, and one male dentist) | BDO, DGZMK, ÖGI, ÖGZMK | 2001, 2020 |
| 52 | Zahnärzte am Ring, Köln | <https://www.zahnaerzte-am-ring.koeln/mundkrebs/> | Germany | Cologne (city) | Private dental practice | Multiple dentists (three female, and two male dentists) | IAAID, DGÄZ, DaCom, DGZMK, DGPro, DGOI | 1992, 2008, 2010, N/R, 2005 |
| 53 | Zahnärzte Dr. Kirchner & Kollegen Köln | <https://www.zahnarzt-kirchner.net/leistungen/zahnvorsorge/mundkrebsvorsorge-velscope/> | Germany | Cologne (city) | Private dental practice | Multiple dentists (two female, and three male dentists) | DGÄZ, DGET, DGZMK, DGSZM, DGFDT, DGI, DGParo | 2006, 2018, 2011, 2021, 2022 |

Supplementary Table A.2. Continued.

| No. | Name of content provider | URL | Country | Location^a^ | Type of provider | Practice setting (sex) | Society membership^b^ | Year of examination^b^ |
| --- | --- | --- | --- | --- | --- | --- | --- | --- |
| 54 | Zahnärzte Dr. Köhler & Partner | <https://www.zahnarzt-dr-koehler.de/1-285--Mundgesundheit.html> | Germany | Pattensen (town) | Private dental practice | Multiple dentists (two female, and one male dentist) | DGFDT, DGI, DGZS, DGKiZ, DGZMK | 2007, 2008, 2022 |
| 55 | Zahnärzte im KantCenter | <https://www.kantcenter.com/mundkrebs-bleibt-oft-unerkannt/> | Germany | Berlin (city) | Private dental practice | Multiple dentists (two female, and two male dentists) | N/R | N/R, N/R, N/R, N/R |
| 56 | Zahnärzte Rheydt | <https://zahnaerzte-rheydt.de/index.php/unsere-leistungen/leistungen3/krebsvorsorge> | Germany | Mönchen-gladbach (city) | Private dental practice | Single dentist (one male dentist) | N/R | N/R |
| 57 | Zahnärztin Claudia Ojeda Kruschel | <https://www.zahnarzt-kruschel.de/?mundhoehlenkrebs> | Germany | Bonn (city) | Private dental practice | Single dentist (one female dentist) | N/R | N/R |
| 58 | Zahnärztin Tanja Siepmann und Zahnarzt Christian Stein | <https://www.zahnarzt-quickborn.de/mundkrebs-fr%C3%BCherkennung> | Germany | Quickborn (town) | Private dental practice | Multiple dentists (one female, and one male dentist) | N/R | N/R, N/R |
| 59 | Zahnärztliche Gemeinschaftspraxis Essen, Dr. med. dent. P. Hentschel / ZA F. Paulun / ZA T. Herdick | <https://www.zahnarztessen.de/diagnostik/mundkrebs-vorsorge/> | Germany | Essen (city) | Private dental practice | Multiple dentists (three female, and four male dentists) | BDIZ EDI, DGI, DGZMK, IGÄM | 1997, N/R, N/R, 1997, 1999, N/R, N/R |

Supplementary Table A.2. Continued.

| No. | Name of content provider | URL | Country | Location^a^ | Type of provider | Practice setting (sex) | Society membership^b^ | Year of examination^b^ |
| --- | --- | --- | --- | --- | --- | --- | --- | --- |
| 60 | Zahnärztliche Gemeinschaftspraxis Dr. Markus Dirheimer & Dr. Elmar Ludwig, mund-pflege.net | <https://mund-pflege.net/operation-chemo-strahlentherapie/> | Germany | Ulm (city) | Private dental practice | Multiple dentists (two male dentists) | N/R | 1999, 2000 |
| 61 | Zahnarztordination Dr. Klaus Charvat | <https://charvat.at/frueherkennungstest-fuer-mundkrebs-in-der-charvat-medlounge/> | Austria | Theresien-feld (rural) | Private dental practice | Single dentist (one male dentist) | ÖGZMK, ÖGP, IMAK, EDA | 1991 |
| 62 | Zahnarztpraxis Adela Deac | <https://www.zahnarztpraxis-deac.de/mundschleimhautzellentest.html> | Germany | Wolfsburg (city) | Private dental practice | Single dentist (one female dentist) | DGZMK | 2006 |
| 63 | Zahnarztpraxis Alstertal, Dr. Esmeralda Pinto dos Santos | <https://www.zahnarztpraxis-alstertal.de/> | Germany | Hamburg (city) | Private dental practice | Multiple dentists (four female, and one male dentist) | DEGUZ, DGÄZ, DGZMK | 2005, 2006, 2019, 2002, N/R |
| 64 | Zahnarztpraxis am Himmelsweg; Dr. Dorothee von Wedel | <https://www.zahnaerzte-tostedt.de/behandlungen/mundh%C3%B6hlenkrebs-vorsorge/> | Germany | Tostedt (town) | Private dental practice | Multiple dentists (five female dentists) | N/R | 2002, N/R, 2008, N/R, N/R |
| 65 | Zahnarztpraxis am Löwenplatz AG; Dr. med. dent. Sara Riedweg | <https://zahnarztloewenplatz.ch/frueherkennung-ist-der-beste-schutz-auch-gegen-mundhoehlen-und-rachenkrebs/> | Switzer-land | Luzern (city) | Private dental practice | Single dentist (one female dentist) | SSO | 2010 |

Supplementary Table A.2. Continued.

| No. | Name of content provider | URL | Country | Location^a^ | Type of provider | Practice setting (sex) | Society membership^b^ | Year of examination^b^ |
| --- | --- | --- | --- | --- | --- | --- | --- | --- |
| 66 | Zahnarztpraxis am Schillerplatz; Nikola Marolov | <https://www.zahnarzt-backnang-mitte.de/> | Germany | Backnang (town) | Private dental practice | Single dentist (one male dentist) | N/R | 2015 |
| 67 | Zahnarztpraxis Dr. Frentz & Kollegen, Inh. Dr. Heike Frentz | <https://www.frentz.de/blog/Zahn%C3%A4rztliches/Tumordiagnostik> | Germany | Stuttgart (city) | Private dental practice | Multiple dentists (six female dentists) | DGZMK, Parodontose-hilfe e.V., GAK, ZAPF, DGÄZ, DGParo, AAE, EAO, PEERS, BDO, DGZ, FVDZ | 1989, 2009, 2010, 2019, 2010, 2024 |
| 68 | Zahnarztpraxis Dr. med. dent Christoph Mülders | <https://zahnarzt-essen.net/> | Germany | Essen (city) | Private dental practice | Single dentist (one male dentist) | DGZMK | 1985 |
| 69 | Zahnarztpraxis Dr. med. dent. Annemarie Stolz | <https://praxis-stolz.at/krebsvorsorge-prevo-check/> | Germany | Volders (rural) | Private dental practice | Multiple dentists (two female dentists) | N/R | 2004, 2022 |
| 70 | Zahnarztpraxis Dr. med. dent. Sabine Langhans, MSc | <https://www.praxislanghans.de/leistungen/krebsfrueherkennung-im-mund-rachenraum/> | Germany | Cologne (city) | Private dental practice | Multiple dentists (two female dentists) | N/R | N/R, N/R |

Supplementary Table A.2. Continued.

| No. | Name of content provider | URL | Country | Location^a^ | Type of provider | Practice setting (sex) | Society membership^b^ | Year of examination^b^ |
| --- | --- | --- | --- | --- | --- | --- | --- | --- |
| 71 | Zahnarztpraxis Dr. med. dent. Sebastian Helm | <https://zahnarztpraxis-helm.de/krebsvorsorge-mundschleimhauterkrankungen.html> | Germany | Mosbach (town) | Private dental practice | Single dentist (one male dentist) | DGZMK, DGI, APW | N/R |
| 72 | Zahnarztpraxis Dr. med. Gerd Bade | <https://www.zahnarzt-hohenschoenhausen.de/1-285--Mundgesundheit.html> | Germany | Berlin (city) | Private dental practice | Single dentist (one male dentist) | N/R | 1983 |
| 73 | Zahnarztpraxis Dr. Regensburger | <https://www.zahnarzt-bogenhausen-muenchen.de/dental-biohealth-center/biologische-zahnheilkunde/krebs/> | Germany | Munich (city) | Private dental practice | Multiple dentists (two male dentists) | APW, DGI, DGÄZ | N/R, 2013 |
| 74 | Zahnarztpraxis Dr. Schröder Stuttgart | <https://www.dr-schroeder-partner.de/zahnarzt-stuttgart-leistungen/mundkrebsvorsorge-stuttgart/> | Germany | Stuttgart (city) | Private dental practice | Multiple dentists (three female, and two male dentists) | GAK, DGAAM, DGI | 1993, 2003, 2018, N/R, N/R |
| 75 | Zahnarztpraxis im Ostseeviertel; Astrid Richter und Kollegen | <https://zahnarztpraxis-richter-berlin.de/leistungen/mundkrebsvorsorge> | Germany | Berlin (city) | Private dental practice | Multiple dentists (five female dentists) | DGI, DGZMK | N/R, N/R, N/R, N/R, N/R |
| 76 | Zahnarztpraxis im Sudturm; Dr. Cornel Lischka & Dr. Philip Ganter | <https://www.zahnarzt-in-gottmadingen.de/mundkrebsfr%C3%BCherkennung/> | Germany | Gottmad-ingen (town) | Private dental practice | Multiple dentists (one female, and two male dentists) | DGZMK, DGParo, DGCZ | 1994, 2009, 2012 |
| 77 | Zahnarztpraxis M. Mattin Nekzai | <https://zahnarztpraxis-nekzai.de/wiki/seltene-orale-erkrankungen/> | Germany | Hamburg (city) | Private dental practice | Single dentist (one male dentist) | Osteology Foundation | 2003 |

Supplementary Table A.2. Continued.

| No. | Name of content provider | URL | Country | Location^a^ | Type of provider | Practice setting (sex) | Society membership^b^ | Year of examination^b^ |
| --- | --- | --- | --- | --- | --- | --- | --- | --- |
| 78 | Zahnarztpraxis Ottensen; Christoph Meyer-Hamme | <https://www.zahnarztpraxis-ottensen-hamburg.de/mundhoehlenkarzinom-krebs-in-der-mundhoehle/> | Germany | Hamburg (city) | Private dental practice | Multiple dentists (two male dentists) | N/R | N/R, N/R |
| 79 | Zahnarztpraxis Stela Xhelili | <https://www.zahnarzt-karli1.de/post/mundkrebs-erkennen-und-fr%C3%BChzeitig-behandeln-alles-was-sie-wissen-m%C3%BCssen> | Germany | Leipzig (city) | Private dental practice | Single dentist (one female dentist) | N/R | N/R |
| 80 | Zahnarztpraxis, Dr. Jan Hinrichsen M.Sc. | <https://www.zahnarzt-kiel-mitte.de/56-285--Mundgesundheit.html> | Germany | Kiel (city) | Private dental practice | Multiple dentists (two male dentists) | N/R | 1994, 2005 |
| 81 | Zahnarztpraxis, Dr. Markus M. Erler | <https://zahnkunde.com/vorsorge/mundkrebs-vorsorge/> | Germany | Siek (rural) | Private dental practice | Single dentist (one male dentist) | DGParo, DGZMK, ITI, FVDZ, VgfZ | 2002 |
| 82 | Zahnarztpraxis, Kerstin Dahlke-Kragelund | <https://www.zahnarztpraxis-grossenwiehe.de/1-285-509-Wei%C3%9Fe-Flecken-im-Mund-k%C3%B6nnen-zu-Mundh%C3%B6hlenkrebs-f%C3%BChren.html> | Germany | Großen-wiehe (rural) | Private dental practice | Single dentist (one female dentist) | N/R | N/R |

Supplementary Table A.2. Continued.

| No. | Name of content provider | URL | Country | Location^a^ | Type of provider | Practice setting (sex) | Society membership^b^ | Year of examination^b^ |
| --- | --- | --- | --- | --- | --- | --- | --- | --- |
| 83 | Zahngesundheit Baden-Baden, Dr. Karsten Kamm, Dr. Torsten Kamm, Dr. Andreas Euchner | <https://zahngesundheit-baden-baden.de/krebsvorsorge-zahnarzt/> | Germany | Baden-Baden (town) | Private dental practice | Multiple dentists (two female, and three male dentists) | AG Keramik, DGÄZ, DGZMK, DGI, DGZS, DGZMS, DGPro, GAK, BDO, DGOI, BDI, ITI, DGET, DGZ | 1996, 1999, 2003, 2008, 2011 |
| 84 | Zahnpraxis Dr. Katharina Hoffmann | <https://www.zahnpraxis-hoffmann.de/spektrum/mundkrebsvorsorge.html> | Germany | Lübeck (city) | Private dental practice | Multiple dentists (one female, and one male dentist) | N/R | 1993, 2021 |
| 85 | Zahnzentrum Rheine Jan-Philipp Trame | <https://www.zahnzentrum-rheine.de/team/blog/11-zahnersatz/28-zahnprothese-nach-tumorbehandlung> | Germany | Rheine (town) | Private dental practice | Multiple dentists (three female, and one male dentist) | BDO, DGOI, DBVE, BAOS, BDA | 2008, 2009, N/R, N/R |
| 86 | Dr. Gumpert GmbH | <https://www.dr-gumpert.de/html/zungenkrebs.html> | Germany | Taunusstein (town) | Information service | N/A | N/A | N/A |
| 87 | MedTech Innovations UG | <https://medrepublic.de/gesunde-zaehne/zungenkrebs_2/> | Germany | Bielefeld (city) | Information service | N/A | N/A | N/A |

Supplementary Table A.2. Continued.

| No. | Name of content provider | URL | Country | Location^a^ | Type of provider | Practice setting (sex) | Society membership^b^ | Year of examination^b^ |
| --- | --- | --- | --- | --- | --- | --- | --- | --- |
| 88 | ONKO-Internetportal | <https://www.krebsgesellschaft.de/onko-internetportal-basis-informationen-krebs-nebenwirkungen-der-therapie-mundschleimhautentz%C3%BCndung.html> | Germany | Berlin (city) | Information service | N/A | N/A | N/A |
| 89 | Selbsthilfenetzwerk Kopf-Hals-M.U.N.D.-Krebs e.V. | <https://www.kopf-hals-mund-krebs.de/kopf-hals-mund-krebs/therapie/> | Germany | Bonn (city) | Information service | N/A | N/A | N/A |
| 90 | AOK-Bundesverband eGbR | <https://www.aok.de/pk/magazin/koerper-psyche/krebs/symptome-und-behandlungsmoeglichkeiten-bei-mundhoehlenkrebs/> | Germany | Berlin (city) | Insurance company | N/A | N/A | N/A |
| 91 | Bundesministerium für Gesundheit (BMG) | <https://gesund.bund.de/mundhoehlenkrebs-rachenkrebs> | Germany | Bonn (city) | Public body | N/A | N/A | N/A |
| 92 | Bundesministerium für Soziales, Gesundheit, Pflege und Konsumentenschutz (BMSGPK), Österreich | <https://www.gesundheit.gv.at/krankheiten/krebs/hals-nasen-ohren/rachenkrebs.html> | Austria | Vienna (city) | Public body | N/A | N/A | N/A |
| 93 | Deutsche Rentenversicherung Rheinland | <https://www.ihre-vorsorge.de/gesundheit/nachrichten/bei-flecken-im-mund-zum-zahnarzt> | Germany | Düsseldorf (city) | Insurance company | N/A | N/A | N/A |

Supplementary Table A.2. Continued.

| No. | Name of content provider | URL | Country | Location^a^ | Type of provider | Practice setting (sex) | Society membership^b^ | Year of examination^b^ |
| --- | --- | --- | --- | --- | --- | --- | --- | --- |
| 94 | Deutscher Zahnversicherungs-Service GmbH & Co.KG | <https://dzvs.de/regelmaessige-zahnarztbesuche-gegen-mundhoehlenkrebs/> | Germany | Hilden (town) | Insurance company | N/A | N/A | N/A |
| 95 | Deutsches Krebsforschungs-zentrum, Krebsinformations-dienst | <https://www.krebsinformationsdienst.de/aktuelles/detail/mangelnde-zahnhygiene-als-krebsrisiko> | Germany | Heidelberg (city) | Society | N/A | N/A | N/A |
| 96 | Getsurance GmbH | <https://getsurance.de/ratgeber/mundhoehlenkrebs-frueherkennung-symptome-und-lebenserwartung/> | Germany | Nuremberg (city) | Insurance company | N/A | N/A | N/A |
| 97 | Initiative proDente e.V. | <https://www.prodente.de/presse/pressemitteilung/weltkrebstag-entzuendung-der-mundschleimhaut-haeufig-unterschaetzte-nebenwirkung.html> | Germany | Cologne (city) | Society | N/A | N/A | N/A |

Supplementary Table A.2. Continued.

| No. | Name of content provider | URL | Country | Location^a^ | Type of provider | Practice setting (sex) | Society membership^b^ | Year of examination^b^ |
| --- | --- | --- | --- | --- | --- | --- | --- | --- |
| 98 | Kassenzahnärztliche Vereinigung Nordrhein | <https://www.zahnpatienten.info/aktuelles/neues-wissenswertes> | Germany | Düsseldorf (city) | Public body | N/A | N/A | N/A |
| 99 | Kassenzahnärztlichen Vereinigung Hamburg und der Zahnärztekammer Hamburg, Das Portal für Patienten und Öffentlichkeit | <https://www.zahnaerzte-hh.de/patientenportal-der-hamburger-zahnaerzte/wissen/fachinformationen-von-a-z/regelmaessig-vorsorgen> | Germany | Hamburg (city) | Public body | N/A | N/A | N/A |
| 100 | Make Sense Campaign; European Head and Neck Society | <https://makesensecampaign.eu/de/informationen-uber-krebs/hpv-und-kopf-und-halskrebs/> | Belgium | Brussels (city) | Public body | N/A | N/A | N/A |
| 101 | Make Sense Kampagne Deutschland; Kongress- und Kulturmanagement GmbH | <https://www.aufklaerung-kopf-hals-krebs.de/Mundh%C3%B6hlenkrebs.html> | Germany | Weimar (town) | Public body | N/A | N/A | N/A |
| 102 | Stiftung Deutsche Krebshilfe | <https://www.krebshilfe.de/informieren/ueber-krebs/krebsarten/krebs-im-mund-kiefer-gesichtsbereich/> | Germany | Bonn (city) | Society | N/A | N/A | N/A |

Supplementary Table A.2. Continued.

| No. | Name of content provider | URL | Country | Location^a^ | Type of provider | Practice setting (sex) | Society membership^b^ | Year of examination^b^ |
| --- | --- | --- | --- | --- | --- | --- | --- | --- |
| 103 | zahn.de; Bayerische Landeszahnärzte-kammer | <https://www.zahn.de/zahn/web.nsf/id/pa_als_krebspatient_zum_zahnarzt.html> | Germany | Munich (city) | Public body | N/A | N/A | N/A |
| 104 | BARMER | <https://www.barmer.de/gesundheit-verstehen/wissen/krankheiten-a-z/mund-und-rachenkrebs-1058276> | Germany | Berlin (city) | Insurance company | N/A | N/A | N/A |
| 105 | 360°zahn, MVZ | <https://www.360gradzahn.de/lexikon/mundhoehlenkarzinom/> | Germany | Düsseldorf (city) | Corporate dental practices | Multiple dentists (fifteen female, and nine male dentists) | N/A | N/A |
| 106 | Alb Fils Klinikum | <https://www.alb-fils-klinikum.de/mund-kiefer-und-plastische-gesichtschirurgie/schwerpunkte-und-krankheitsbilder/gut-und-boesartige-tumoren-im-kopf-hals-bereich/> | Germany | Göppingen (town) | Private hospital group | Multiple dentists (two female, and seven male dentists) | N/A | N/A |
| 107 | Alfried Krupp von Bohlen und Halbach Krankenhaus gemeinnützige GmbH | <https://www.krupp-krankenhaus.de/hno-heilkunde-kopf-und-hals-chirurgie/leistungsspektrum/kopf-hals-tumoren.html> | Germany | Essen (city) | Private hospital group | N/A | N/A | N/A |
| 108 | AllDent Zahnzentrum München GmbH (Standort München Hauptbahnhof) | <https://www.alldent-zahnzentrum.de/leistungen/fachbegriffs-lexikon/begriff/mundhoehlenkrebs-karzinom.html> | Germany | Munich (city) | Corporate dental practice | Multiple dentists (twenty-eight female, and nine male dentists) | N/A | N/A |

Supplementary Table A.2. Continued.

| No. | Name of content provider | URL | Country | Location^a^ | Type of provider | Practice setting (sex) | Society membership^b^ | Year of examination^b^ |
| --- | --- | --- | --- | --- | --- | --- | --- | --- |
| 109 | BG Klinikum Unfallkrankenhaus Berlin | <https://www.bg-kliniken.de/unfallkrankenhaus-berlin/fachbereiche/detail/kopf-hals-tumorzentrum/> | Germany | Berlin (city) | Private hospital group | N/A | N/A | N/A |
| 110 | Charité Comprehensive Cancer Center | <https://cccc.charite.de/fuer_patientinnen_interessierte/organkrebszentren/kopf_und_hals_tumore> | Germany | Berlin (city) | University hospital | N/A | N/A | N/A |
| 111 | Daniel Haensch, Zahnarzt (Deutschland) DentsPro GmbH | <https://www.dentspro.de/news/335-mundhoehlenkrebs> | Germany | Berlin (city) | Corporate dental practices | Multiple dentists (five female, and ten male dentists) | N/A | N/A |
| 112 | Dental Team Oberbayern MVZ GmbH | <https://dental-team-oberbayern.de/leistungen/krebsvorsorge/> | Germany | Mühldorf am Inn (town) | Corporate dental practice | Multiple dentists (one female, and three male dentists) | N/A | N/A |
| 113 | dentaloft MVZ GmbH; Dr. Peter Schütte, Christiane Kunze | [https://www.dentaloft.de//vorsorge/mundhoehlenkrebs](https://www.dentaloft.de/vorsorge/mundhoehlenkrebs) | Germany | Frankfurt am Main (city) | Corporate dental practice | Multiple dentists (four female, and three male dentists) | N/A | N/A |
| 114 | Die Zahnärzte Dres. Fuchs & Kollegen GmbH | <https://www.die-zahnaerzte.de/behandlungen/mundkrebs-frueherkennung> | Germany | Constance (town) | Corporate dental practice | Multiple dentists (three female, and one male dentist) | N/A | N/A |
| 115 | Dr. med. Adrian Staab Zentrum für Strahlentherapie | <https://strahlentherapie-staab.de/erkrankungen/kopf-hals-tumoren/> | Germany | Bad Kreuznach (town) | Corporate practice | N/A | N/A | N/A |

Supplementary Table A.2. Continued.

| No. | Name of content provider | URL | Country | Location^a^ | Type of provider | Practice setting (sex) | Society membership^b^ | Year of examination^b^ |
| --- | --- | --- | --- | --- | --- | --- | --- | --- |
| 116 | FD Holding GmbH, fair-doctors.de | <https://fair-doctors.de/mundkrebs-symptome-und-behandlung/> | Germany | Cologne (city) | Corporate practice | N/A | N/A | N/A |
| 117 | Gesundheit Nord gGmbH Klinikverbund Bremen | <https://www.gesundheitnord.de/kbm/zertifiziertes-onkologisches-zentrum-/-zertifizierte-organkrebszentren/kopf-hals-tumorzentrum-bremen-mitte.html> | Germany | Bremen (city) | Private hospital group | N/A | N/A | N/A |
| 118 | Klinikum Lüneburg | <https://www.klinikum-lueneburg.de/strahlentherapie-und-radioonkologie/strahlentherapie-bei-tumoren-in-der-kopf-hals-region> | Germany | Lunenburg (town) | Private hospital group | N/A | N/A | N/A |
| 119 | Medias Klinikum GmbH & Co. KG | <https://www.medias-klinikum.de/home/f%C3%BCr-patienten/krebsarten/kopf-hals-tumore/> | Germany | Burghausen (town) | Private hospital group | N/A | N/A | N/A |
| 120 | Medizinisches Versorgungszentrum Zahnmedizin Essen-Zentrum | <https://www.berater-zahnaerzte.de/praxis/moderne-technik/mundkrebsdiagnostik> | Germany | Essen (city) | Corporate practice | Multiple dentists (one female, and four male dentists) | N/A | N/A |
| 121 | MKG Chirurgie Allee Center Hamm MVZ GmbH | <https://www.mkg-chirurgie-hamm.de/mundhoehlenkrebs-diagnostik-und-behandlung/> | Germany | Hamm (city) | Corporate practice | Multiple dentists (five male dentists) | N/A | N/A |

Supplementary Table A.2. Continued.

| No. | Name of content provider | URL | Country | Location^a^ | Type of provider | Practice setting (sex) | Society membership^b^ | Year of examination^b^ |
| --- | --- | --- | --- | --- | --- | --- | --- | --- |
| 122 | MVZ Limbecker Platz Essen GmbH, Die Zahnärzte am Limbecker Platz | <https://www.zahnarzt-essen-zentrum.de/leistungen/krebsvorsorge/> | Germany | Essen (city) | Corporate practice | Multiple dentists (ten female, and four male dentists) | N/A | N/A |
| 123 | Kaiserberg Zahnärztezentrum | <https://www.kaiserberg-zmvz.de/leistungen/mundkrebs-vorsorge/> | Germany | Duisburg (city) | Corporate practice | Multiple dentists (two female, and one male dentist) | N/A | N/A |
| 124 | MVZ MCLINIC Interdisziplinäres Facharztzentrum München GmbH | <https://www.mclinic.de/fachbereiche/mface/gesicht-und-kopf/kopf-hals-tumoren/> | Germany | Munich (city) | Corporate practice | Multiple dentists (one female, and five male dentists) | N/A | N/A |
| 125 | MVZ Zahnärzte am Werk | <https://www.zahnarzt-leverkusen.de/behandlungen/strahlenschutzschiene> | Germany | Leverkusen (city) | Corporate practice | Multiple dentists (three male dentists) | N/A | N/A |
| 126 | Universitäts Spital Zürich | <https://www.usz.ch/fachbereich/mund-kiefer-gesichtschirurgie/> | Switzer-land | Zurich (city) | University hospital | Multiple dentists (eight female, and fourteen male dentists) | N/A | N/A |
| 127 | Universitätsklinikum Düsseldorf | [https://www.uniklinik-duesseldorf.de/patienten-besucher/klinikeninstitutezentren/klinik-fuer-mund-kiefer-und-plastische](https://www.uniklinik-duesseldorf.de/patienten-besucher/klinikeninstitutezentren/klinik-fuer-mund-kiefer-und-plastische-) | Germany | Düsseldorf (city) | University hospital | N/R | N/A | N/A |
| 128 | Universitätsklinikum Freiburg | <https://www.uniklinik-freiburg.de/cccf/onkologisches-spitzenzentrum/kopf-hals-tumorzentrum.html> | Germany | Freiburg (city) | University hospital | Multiple dentists (thirteen female, and fourteen male dentists) | N/A | N/A |

Supplementary Table A.2. Continued.

| No. | Name of content provider | URL | Country | Location^a^ | Type of provider | Practice setting (sex) | Society membership^b^ | Year of examination^b^ |
| --- | --- | --- | --- | --- | --- | --- | --- | --- |
| 129 | Universitätsklinikum Heidelberg | <https://www.klinikum.uni-heidelberg.de/erkrankungen/boesartige-tumoren-des-kopf-hals-bereich-201186> | Germany | Heidelberg (city) | University hospital | N/R | N/A | N/A |
| 130 | Universitätsklinikum Mannheim GmbH | <https://www.umm.de/hals-nasen-ohren-klinik/leistungsspektrum/kopf-hals-tumore/> | Germany | Mannheim (city) | University hospital | N/R | N/A | N/A |
| 131 | Universitätsklinikum Tübingen | <https://www.medizin.uni-tuebingen.de/de/das-klinikum/einrichtungen/kliniken/radioonkologie/patienten/kopf-hals-region> | Germany | Tübingen (town) | University hospital | N/R | N/A | N/A |
| 132 | Westdeutsches Protonentherapie-zentrum Essen | <https://www.wpe-uk.de/eine-gute-vorbereitung-ist-essenziell/> | Germany | Essen (city) | Private hospital group | N/A | N/A | N/A |
| 133 | zahnarztzentrum.ch AG | <https://zahnarztzentrum.ch/wie-laesst-sich-mundhoehlenkrebs-erkennen> | Switzer-land | Zurich (city) | Corporate practice | N/A | N/A | N/A |
| 134 | Zahnzentrum am Mendelssohnplatz, H.-J. Werner & Kollegen | <https://www.zahnarzt-werner-ka.de/krebsvorsorge/> | Germany | Karlsruhe (city) | Corporate practice | Multiple dentists (four male dentists) | N/A | N/A |

^a^ Content provider’s location (rural, town [<100,000 inhabitants], or city [≥100,000 inhabitants]); ^b^ according to public website; DGI, German Association of Oral Implantology; DGZMK, German Society of Dentistry and Oral Medicine; GZFA, Society for Oral Health, Function, and Aesthetics; DGET, German Association for Dental Traumatology; DGMKG, German Association of Oral and Maxillofacial Surgery; DGParo, German Society of Periodontology; FVDZ, Free Association of German Dentists; DGÄZ, German Association of Esthetic Dentistry; APW, Academy Practice and Science; VDZE, Association of Certified German Endodontists; DGPro, German Society for Prosthetic Dentistry and Biomaterials; BDO, German Professional Association of Oral Surgeons; BDIZ EDI, European Association of Dental Implantologists; DGFDT, German Society for Functional Diagnostics and Therapy; DGZ, German Society of Conservative Dentistry; DGOI, German Society of Oral Implantology; ITI, International team for implantology; NLI, North German Association of Implantology; DGCZ, German Society for Computer-Assisted Dentistry; DGZI, German Society for Dental Implantology; PZVD, Private Dentists' Association of Germany; DGZS, German Society of Dental Sleep Medicine; DGZH, German Society for Dental Hypnosis; DGKiZ, German Society of Paediatric Dentistry; MEG, Milton Erickson Society for Clinical Hypnosis; DGBT, German Society for Aesthetic Botulinum and Filler Therapy; DEGUZ, German Society for Environmental Dental Medicine; ZVFFM, Dental Association of Frankfurt am Main, established in 1863; DGL, German Society for Laser Dentistry; DGM, German Society for Mesotherapy; GSAAM, German Society for Prevention and Anti-Aging Medicine; DZOI, German Center for Oral Implantology; DGAZ, German Society for Geriatric Dentistry; ÖGP, Austrian Society of Periodontology; DEGUM, German Society for Ultrasound in Medicine; GTÜM, Society for Diving and Hyperbaric Medicine; DGMM, man Society for Manual Medicine; DGHNOKC, German Society for Otorhinolaryngology Head and Neck Surgery; DGAAM, German Society for Acupuncture and Auriculomedicine; AKI, Working Group on Implantology; GAK, Gnathological Working Group Stuttgart; DAA, German Academy of Acupuncture; DZV, German Dentists' Association; SSO, Swiss Dental Association; ÖGI; Austrian Society of Implantology; ÖGZMK, Austrian Society of Dentistry, Oral and Maxillofacial Medicine; IAAID, International Academy for Advanced Interdisciplinary Dentistry; DaCom, German Academy of Cranial and Orofacial Medicine; DGSZM, Society for Sports Dentistry; IGÄM, International Society for Aesthetic Medicine and Dentistry; EDA, European Dental Association; IMAK, International Medical Society for Applied Kinesiology; ZAPF, Dental Working Group for Practice Management and Continuing Education; AAE, American Association of Endodontists; EAO, European Association for Osseointegration; PEERS, Platform Exchange Education Research Science; VgfZ, Contractual Association of Independent Dentists in Schleswig-Holstein; BDI, Professional Association of German Internists; DBVE, German National Association of Anaplastologists; BAOS, British Association of Oral Surgery; BDA, British Dental Organisation; N/A, not applicable; N/R, not reported.

Supplementary Table A.3. Characteristics of included YouTube videos with eHealth information for HNC patients in dental context.

| No. | Name of content provider | URL | Country | Location^a^ | Type of provider | Society membership^b^ | Year of examination^b^ | Upload date | Duration (in minutes) | Number of likes | Number of comments |
| --- | --- | --- | --- | --- | --- | --- | --- | --- | --- | --- | --- |
| 1 | Universitäts-klinikum Freiburg | <https://www.youtube.com/watch?v=dvPjth1c4tw> | Germany | Freiburg (city) | University hospital | N/A | N/A | March 11, 2021 | 13:26 | 0 | 0 |
| 2 | LMU Klinikum | <https://www.youtube.com/watch?v=dY2_Bim1iYw> | Germany | Munich (city) | University hospital | N/A | N/A | May 22, 2023 | 05:36 | 1 | 0 |
| 3 | Selbsthilfe- netzwerk Kopf-Hals-M.U.N.D.-Krebs e.V. | <https://www.youtube.com/watch?v=sicpDo1nkYE> | Germany | Bonn (city) | Information service | N/A | N/A | July 13, 2022 | 05:51 | 11 | 3 |
| 4 | Klinikum Dortmund | <https://www.youtube.com/watch?v=W0eL5hYhWmM> | Germany | Dortmund (city) | Private hospital group | N/A | N/A | February 21, 2018 | 10:58 | 111 | 5 |
| 5 | health tv | <https://www.youtube.com/watch?v=sidHI4RVIPA> | Germany | Königstein im Taunus (town) | Information service | N/A | N/A | April 06, 2022 | 05:17 | 65 | 7 |
| 6 | Selbsthilfe-netzwerk Kopf-Hals-M.U.N.D.-Krebs e.V. | <https://www.youtube.com/watch?v=9Hw_3iIeaDw> | Germany | Bonn (city) | Information service | N/A | N/A | July 7, 2022 | 06:17 | 27 | 0 |

Supplementary Table A.3. Continued.

| No. | Name of content provider | URL | Country | Location^a^ | Type of provider | Society membership^b^ | Year of examination^b^ | Upload date | Duration (in minutes) | Number of likes | Number of comments |
| --- | --- | --- | --- | --- | --- | --- | --- | --- | --- | --- | --- |
| 7 | Dr. Jung Zahnklinik | <https://www.youtube.com/watch?v=49nvXoMsNUU> | Germany | Frankfurt am Main (city) | Private dental practice | DGZMK, DGI, BDIZ, NagP, DGCZ, DGÄZ, DGL, DÄGfA | 1989, 2022, N/R, N/R | March 05, 2018 | 04:56 | 6 | 1 |
| 8 | mydenttv | <https://www.youtube.com/watch?v=IUvJ-44SR0k> | Germany | Mainz (city) | Public body | N/A | N/A | April 24, 2012 | 08:58 | 0 | 0 |
| 9 | Health Cerlerates | <https://www.youtube.com/watch?v=Apiceds0Xh4> | Germany | Hamburg (city) | Information service | N/A | N/A | December 13, 2023 | 07:19 | 7 | 0 |
| 10 | Kiefergelenk-Zentrum | <https://www.youtube.com/watch?v=QZxkFPNyYbQ> | Switzerland | Zurich (city) | Private dental practice | SGMKG, DKG, DÖSAK, EACR | 1998 | May 10, 2023 | 04:29 | 242 | 3 |
| 11 | Thalamus | <https://www.youtube.com/watch?v=7RqOBEP76Yo> | Canada | Toronto (city) | Information service | N/A | N/A | October 12, 2021 | 01:53 | 0 | 0 |
| 12 | Denta1 Clinic - Dr. Stefan Helka | <https://www.youtube.com/watch?v=QrLlGcBm8N0> | Germany | Herne (city) | Corporate practice | N/R | 2008, 2017, 2009, N/R, 2017, N/R, 2020, N/R, N/R, N/R, N/R, N/R | August 09, 2021 | 13:06 | 1,651 | 197 |

Supplementary Table A.3. Continued.

| No. | Name of content provider | URL | Country | Location^a^ | Type of provider | Society membership^b^ | Year of examination^b^ | Upload date | Duration (in minutes) | Number of likes | Number of comments |
| --- | --- | --- | --- | --- | --- | --- | --- | --- | --- | --- | --- |
| 13 | Presseportal Videos | <https://www.youtube.com/watch?v=Xc0xpD16rGQ> | Germany | Hamburg (city) | Information service | N/A | N/A | May 31, 2022 | 01:03 | 1 | 0 |
| 14 | medumio - Wissen, was hilft. Wissen, das hilft. | <https://www.youtube.com/watch?v=qsWYPhHf1T4> | Germany | Berlin (city) | Information service | N/A | N/A | March 23, 2023 | 10:57 | 11 | 3 |
| 15 | Städtisches Klinikum Karlsruhe gGmbH | <https://www.youtube.com/watch?v=7tqjpewCRig> | Germany | Karlsruhe (city) | Private hospital group | N/A | N/A | November 20, 2019 | 11:55 | 164 | 24 |
| 16 | AOK - Der Gesundheits-kanal | <https://www.youtube.com/watch?v=iO1kB5MGTg0> | Germany | Berlin (city) | Insurance company | N/A | N/A | August 21, 2023 | 02:46 | 2,013 | 287 |
| 17 | Charité - Universitätsmedizin Berlin | <https://www.youtube.com/watch?v=DqtMPSbQD2I> | Germany | Berlin (city) | University hospital | N/A | N/A | December 05, 2023 | 03:26 | 20 | 1 |
| 18 | Universitäts-klinikum Freiburg | <https://www.youtube.com/watch?v=N04JZGLP6aI> | Germany | Freiburg (city) | University hospital | N/A | N/A | February 19, 2023 | 04:31 | 0 | 23 |
| 19 | Apotheken Umschau | <https://www.youtube.com/watch?v=yJhDDxV6LHY> | Germany | Baierbrunn (rural) | Information service | N/A | N/A | June 07, 2023 | 03:37 | 25 | 2 |

Supplementary Table A.3. Continued.

| No. | Name of content provider | URL | Country | Location^a^ | Type of provider | Society membership^b^ | Year of examination^b^ | Upload date | Duration (in minutes) | Number of likes | Number of comments |
| --- | --- | --- | --- | --- | --- | --- | --- | --- | --- | --- | --- |
| 20 | Charité – Universitäts-medizin Berlin | <https://www.youtube.com/watch?v=MbefN8D8OFk> | Germany | Berlin (city) | University hospital | N/A | N/A | July 11, 2024 | 05:43 | 14 | 0 |
| 21 | Hirslanden-Gruppe | <https://www.youtube.com/watch?v=XKWjbGjEB-8> | Switzerland | Glattpark (town) | Private hospital group | N/A | N/A | April 20, 2018 | 01:09 | 3 | 0 |
| 22 | Thea Lingohr | <https://www.youtube.com/watch?v=xVu3WnK1YlY> | Germany | Cologne (city) | Private dental practice | N/R | N/R, N/R, N/R | April 28, 2020 | 01:11 | 3 | 1 |
| 23 | Hirslanden-Gruppe | <https://www.youtube.com/watch?v=gOcqoRxGEwI> | Switzerland | Glattpark (town) | Private hospital group | N/A | N/A | October 03, 2017 | 06:07 | 33 | 0 |
| 24 | Zahnarzt Dr. Butz & Partner, München | <https://www.youtube.com/watch?v=BcV3D1U2U-g> | Germany | Munich (city) | Private dental practice | DGI, DGZMK, DGET, BDO, DGMKG | 2007, 2004, 2007, 2006, 2022, 2013, 2011 | August 18, 2020 | 01:39 | 1 | 0 |
| 25 | Drstephan-anders | <https://www.youtube.com/watch?v=W1-vHwF5DrA&t=1s> | Germany | Bad Aibling (town) | Private dental practice | N/R | N/R, N/R | May 03, 2023 | 03:52 | 0 | 0 |

Supplementary Table A.3. Continued.

| No. | Name of content provider | URL | Country | Location^a^ | Type of provider | Society membership^b^ | Year of examination^b^ | Upload date | Duration (in minutes) | Number of likes | Number of comments |
| --- | --- | --- | --- | --- | --- | --- | --- | --- | --- | --- | --- |
| 26 | Krebs-informations-dienst des Deutschen Krebs-forschungs-zentrums | <https://www.youtube.com/watch?v=h6iiEM9IuU4> | Germany | Heidelberg (city) | Society | N/A | N/A | February 19, 2020 | 01:59 | 6 | 0 |

^a^ Content provider’s location (rural, town [<100,000 inhabitants], or city [≥100,000 inhabitants]); ^b^ according to public website; DGI, German Association of Oral Implantology; DGZMK, German Society of Dentistry and Oral Medicine; DGÄZ, German Association of Esthetic Dentistry; DGCZ, German Society for Computer-Assisted Dentistry; DGL, German Society for Laser Dentistry; BDIZ EDI, European Association of Dental Implantologists; DÄGfA, German Medical Association for Acupuncture; NagP, New Periodontology Working Group; SGMKG, Swiss Society of Oral and Maxillofacial Surgery; DKG, German Cancer Society; DÖSAK, German-Austrian-Swiss Working Group on Tumors of the Jaw and Facial Regio; EACR, European Association for Cancer Research; DGET, German Association for Dental Traumatology; DGMKG, German Association of Oral and Maxillofacial Surgery; BDO, German Professional Association of Oral Surgeons; N/A, not applicable; N/R, not reported.
